# Obesity susceptibility loci in Qataris, a highly consanguineous Arabian population

**DOI:** 10.1186/s12967-015-0459-3

**Published:** 2015-04-13

**Authors:** Sara Tomei, Ravinder Mamtani, Rashid Al Ali, Naser Elkum, Maryam Abdulmalik, Awatef Ismail, Sohaila Cheema, Hekmat A Rouh, Idil I Aigha, Fatima Hani, Sura Al-Samraye, Mona Taher Aseel, Nada El Emadi, Azza Al Mujalli, Ahmed Abdelkerim, Siddik Youssif, Andrea Worschech, Emad El Sebakhy, Ramzi Temanni, Vineesh Khanna, Ena Wang, Dhanya Kizhakayil, Al-Anood Al-Thani, Mohammed Al-Thani, Albert Lowenfels, Francesco M Marincola, Javaid Sheikh, Lotfi Chouchane

**Affiliations:** Department of Genetic Medicine, Laboratory of Genetic Medicine & Immunology, Weill Cornell Medical College in Qatar, Al Luqta Street, Qatar Foundation, Education City, Doha, Qatar PO 24144,; Sidra Medical and Research Center, Research Branch, Al Nasr Tower, Al Corniche Street, Qatar Foundation, Doha, Qatar PO 26999,; Global and Public Health Department, Weill Cornell Medical College in Qatar, Doha, Qatar; Primary Health Care Corporation, Doha, Qatar; Supreme Council of Health, Doha, Qatar; New York Medical College, New York, USA; Dean’s Office, Weill Cornell Medical College in Qatar, Doha, Qatar

**Keywords:** Single Nucleotide Polymorphism (SNP), Genotyping, Qatar, Obesity, Body mass index

## Abstract

**Objectives:**

In Qataris, a population characterized by a small size and a high rate of consanguinity, between two-thirds to three-quarters of adults are overweight or obese. We investigated the relevance of 23 obesity-related loci in the Qatari population.

**Methods:**

Eight-hundred-four individuals assessed to be third generation Qataris were included in the study and assigned to 3 groups according to their body mass index (BMI): 190 lean (BMI < 25 kg/m^2^); 131 overweight (25 kg/m^2^ ≤ BMI < 30 kg/m^2^) and 483 obese (BMI ≥ 30 kg/m^2^). Genomic DNA was isolated from peripheral blood and genotyped by TaqMan.

**Results:**

Two loci significantly associated with obesity in Qataris: the TFAP2B variation (rs987237) (A allele versus G allele: chi-square = 10.3; P = 0.0013) and GNPDA2 variation (rs10938397) (A allele versus G allele: chi-square = 6.15; P = 0.013). The TFAP2B GG genotype negatively associated with obesity (OR = 0.21; P = 0.0031). Conversely, the GNDPA2 GG homozygous genotype associated with higher risk of obesity in subjects of age < 32 years (P = 0.0358).

**Conclusion:**

We showed a different genetic profile associated with obesity in the Qatari population compared to Western populations. Studying the genetic background of Qataris is of primary importance as the etiology of a given disease might be population-specific.

**Electronic supplementary material:**

The online version of this article (doi:10.1186/s12967-015-0459-3) contains supplementary material, which is available to authorized users.

## Introduction

Obesity is a medical condition characterized by an accumulation of excess of body fat, which eventually results in a reduced life expectancy and/or increased health problems. Obesity frequently associates with dyslipidemia, hypertension, insulin resistance, type 2 diabetes mellitus (T2DM) and cardiovascular diseases [1]. Adipocyte-specific metabolic differences have been described in literature [2]. The incidence and prevalence of obesity are rising rapidly to epidemic proportions both in the industrial world and worldwide. Obesity results from an imbalance between energy intake and energy expenditure. As a multifactorial disease, its onset is due to the interplay between environmental and genetic factors. Environmental risk factors include the use of dense fast food, sugar-sweetened beverages, large portion sizes, a sedentary behavior, physical inactivity and shortened sleep period [3]. In parallel, genetic factors also contribute to the etiology of obesity [4]. Although cases of monogenic obesity (caused by mutations in single genes) have been described in literature [5], obesity in mainly polygenic. Polygenic obesity results from a complex interaction between multiple genes and the environment. Although these genes do not directly cause obesity, they can influence obesity development in certain environments and/or in concert with other genetic alterations. Several GWAS studies have identified Single Nucleotide Polymorphisms (SNPs) associated to obesity and fat distribution [6-10].

While current studies provide data about obesity risk factors, they have been mainly focused on Western populations and their conclusions do not necessarily apply to populations with a different genetic background. Clearly, additional research is warranted to better understand obesity risk factors in populations disproportionately affected by obesity like the Qatari population.

In Qataris, a population characterized by a very small size with a particularly high rate of consanguinity, which can reach 54% due to frequent first-degree cousin unions [11], more than 70% of the adult population is overweight or obese [12]. Such a high rate of obesity has been attributed to the lifestyle changes associated with the discovery of oil and the subsequent increase in wealth. Urbanization has occurred rapidly in Qatar and has been accompanied by the acquisition of a sedentary lifestyle, which may have contributed to the increased obesity incidence and prevalence. Recent GWAS studies have identified genetic factors predisposing/protecting to obesity; however, it is not well known whether these genetic markers confer similar or different risks across people of different ancestry. Although an uneven distribution of disease-associated alleles between populations of different ancestry has been shown for recessive Mendelian disorders [13,14], whether this phenomenon applies also to complex diseases is not yet well understood. Markers identified by GWAS studies may confer a risk, which can vary according to the different ancestry of the populations under study. This phenomenon can be due to the variability of allelic frequencies and/or to differences in linkage disequilibrium (LD) between the identified variants and the true functional variants that underlie disease risk [15,16]. In this regard, a recent study interestingly showed that the genetic risk for type 2 diabetes and pancreatic cancer decreased as humans migrated toward East Asia, highlighting a genetic risk differentiation of multifactorial diseases across human populations [17].

In this report we selected 23 loci found associated to obesity in previous studies and tested them in the Qatari population. This question is particularly relevant considering that little is known about the genetic background of the Arabian populations. When selecting the SNPs to test, we aimed at choosing the ones mapping within or in proximity of well documented obesity genes (as FTO and MC4R, for instance) and as representative of the obesity status (both in terms of BMI and waist circumference). These 23 SNPs included 19 loci associated to BMI (of which 11 were recently identified) [9,10,18-21] and 4 loci associated to body weight and waist circumference [9,18,22].

The phenotype/genotype interaction was also investigated by analyzing the association of the obesity SNPs with phenotypic and clinical parameters.

The genetic investigation of diversity in human physical characteristics has a rich history and several ancestry informative markers (AIM), defined as human polymorphisms that exhibit substantially different frequencies among populations, have been identified and proved able to discriminate human populations [23]. However, the weight played by variations associated to multifactorial diseases (like obesity) in discriminating human populations has been poorly investigated.

By comparing the 23 SNPs genotyping data from our cohort of Qatari lean subjects with the data from the 1000Genomes Project, we show here that such disease-related loci are able to differentiate ethnically different human populations to a lesser extent when applied to Qataris, most likely due to their heterogeneity as Qataris derive from populations of different genetic background.

## Materials and methods

### Subjects’ recruitment

A total of 804 Qatari subjects with no known familial relationship were recruited from the health clinic at Hamad Medical Corporation Hospital, Doha, Qatar, on a voluntary basis. The study was approved by the Research Ethics Committee of Hamad Medical Corporation and by Institutional Review Board of Weill Cornell Medical College in Qatar. All the subjects who agreed to participate in this study gave informed consent prior to their inclusion in the study. Only individuals assessed to be third generation Qataris (as reported by questionnaires) were included in the study. Individuals were assigned to three different groups according to their body mass index (BMI), as following:

- 190 lean (BMI < 25 kg/m^2^);
- 131 overweight (25 kg/m^2^ ≤ BMI < 30 kg/m^2^);
- 483 obese (BMI ≥ 30 kg/m^2^).

For the association purposes, only lean and obese group were included in the analysis. The lean group included 124 (65%) female and 66 (35%) male subjects. The obese group included 378 (78%) female and 105 (22%) male subjects. The average ages were 31.36 and 48.06 for the lean and obese groups, respectively.

### Phenotypic and clinical variables assessment

WC (waist circumference, cm) was measured midway between the lowest rib and the superior border of the iliac crest on standing subjects by a single examiner using an inelastic tape. BMI was calculated as weight (kg) divided by the squared height (m^2^). A questionnaire solicited information about age, gender, parent and grandparent ethnicity, sleep hours and the presence of sleep disorders. The presence or absence of cardiovascular diseases, T2DM and cholesterol disorders was also recorded.

Data was obtained from as many subjects as possible. In some cases only partial data was available as some subjects refused to answer all the items in the questionnaires.

### DNA isolation

A 10 mL blood sample was collected in sterile sodium heparin collection tubes for each individual recruited in the study. Genomic DNA was isolated from peripheral blood samples using QIAamp® DNA Blood Maxi Kit according to the manufacturer’s protocol (Qiagen, Valencia, CA). DNA quality and quantity were estimated using Nanodrop (Thermo Scientific, Waltham, Massachusetts, USA).

### Genotyping

Twenty-three SNPs reported as obesity-related in previous studies were selected and genotyped using TaqMan technology. TaqMan pre-designed SNP genotyping assays were chosen from Applied Biosystems website (https://products.appliedbiosystems.com/ab/en/US/adirect/ab). Quantitative Real-Time PCR (qPCR) was performed in a final volume of 25 uL containing the TaqMan® Universal PCR Master Mix (without AmpErase® UNG), the SNP Genotyping Assay Mix (SNP assay-specific) and 10–50 ng of genomic DNA. Each pre-formulated SNP Genotyping Assay Mix included the forward and reverse primers (900 nM final concentration), and the VIC- and 6FAM-conjugated probes (200 nM final concentration) which detected the two alternative alleles, respectively. QPCR was carried out on a 7500 Fast Real-Time PCR System machine (Applied Biosystems, Grand Island, NY). Each PCR reaction used distilled water instead of DNA as negative control. The thermal cycling conditions were as follows: initial denaturing at 95°C for 10 min, 40 cycles of 92°C for 15 s and 60°C for 1 min.

After PCR amplification, an endpoint plate read was performed. Fluorescence measurements obtained during the plate read were used by the Sequence Detection System (SDS) Software to plot fluorescence (Rn) values based on the signals from each single plate well. Results were plotted on a two-dimensional scatter plot of the major allele versus the minor allele. Genotyping calls were assessed based on the allele discrimination plots and manually reviewed by looking at the single amplification plots.

### Statistical analyses

We compared the baseline characteristics of the participants using analysis of variance tests (ANOVA) for continuous variables. Categorical variables were analyzed using the chi-square test. Mean BMI values were estimated within each group of homozygous reference, heterozygous, and homozygous variant genotypes for each SNP. The mean BMI estimates were adjusted by age and gender using analysis of covariance (ANCOVA) approach. Multivariable logistic regression analysis was performed to estimate odds ratios (ORs) adjusted for covariates and to assess the predictive effect of each SNP on risk of developing obesity. Data is reported as mean ± standard deviation (SD) and range, unless stated otherwise. All statistical assessments were two-sided and considered to be significant when P-value < 0.05. All analyses were performed using SAS (version 9.4; SAS Institute, Cary, NC). Hardy-Weinberg equilibrium (HWE) was tested by chi-square.

Genotyping data for the 23 SNPs under study were downloaded from the 1000Genomes Project website (http://www.1000genomes.org/) from Africans (61 ASW, American of African Ancestry in South West USA; 97 LWK, Luhya in Webuye, Kenya; 88 YRI, Yoruba in Ibadan, Nigeria), Americans (60 CLM, Colombians from Medellin, Colombia; 66 MXL, Mexican Ancestry from Los Angeles, USA; 55 PUR, Puerto Ricans from Puerto Rico), Asians (97 CHB, Han Chinese in Bejing, China; 100 CHS, Southern Han Chinese; 89 JPT, Japanese in Tokyo, Japan), Europeans (85 CEU, Utah Residents (CEPH) with Northern and Western European ancestry; 93 FIN, Finnish in Finland; 89 GBR, British in England and Scotland; 14 IBS, Iberian population in Spain; 98 TSI, Toscani in Italy). In the American MXL population, offspring genotypes (n = 2) were removed to analyze unrelated subjects. Unphased genotyping data from the 1000Genomes Project and healthy Qatari lean subjects was used for Principal Component Analysis (PCA) visualization. PCA plots are presented based on Partek Genomic Suite software. PCA was performed by using the “Super Population Codes” for graphical purposes, namely, AFR for Africans, ASN for Asian, EUR for Europeans, AMR for Americans. Qatari lean subjects were called QAR when performing PCA.

## Results

### General characteristics of Qatari populations

Table 1 shows the phenotypic and clinical characteristics of lean, overweight and obese subjects. As expected, the presence of CVD, T2DM and cholesterol disorders was significantly higher in obese compared to lean subjects. WC was also significantly associated to the obese group compared to the lean group. A nominal difference was found in sleeping hours between obese and lean, with the former showing a sleep trend toward “less than 8 hours”.

**Table 1:** **Phenotypic and clinical parameters of lean, overweight and obese Qatari subjects**

**Phenotypic/clinical parameter**	**Lean**	**Overweight**	**Obese**	**P-value (comparing obese vs. lean)**
Sex (M/F)	66/124	44/87	105/378	0.0008
Age (y ± SD)	31.37 ± 13.5	35.93 ± 13.6	48.06 ± 13.4	<0.0001
BMI (kg/m^2^ ± SD)	22.2 ± 2.1	27.6 ± 1.4	36.2 ± 5.6	<0.0001
WC (cm ± SD)	78.7 ± 9.1	91.8 ± 7.4	106.1 ± 13.0	<0.0001
Sleep hours (A/B/C)	37/93/25	35/76/18	82/123/27	0.04
Sleep disorders (Y/N)	42/101	37/87	77/151	0.42
CVD (Y/N)	6/150	24/103	74/159	<0.0001
T2DM (Y/N)	4/152	23/105	68/162	<0.0001
Cholesterol disorder (Y/N)	8/147	27/100	89/144	<0.0001

M, male; F, female; SD, standard deviation; BMI, body mass index; WC, waist circumference; Sleep hours (A/B/C), A, less than 6 hours, B, between 6 and 8 hours, C, more than 8 hours; Y, yes (presence); N, no (absence); CVD, cardiovascular diseases; T2DM, type 2 diabetes mellitus.

P values were calculated comparing obese vs. lean only, Fisher and Student’s *t* tests were applied as appropriate.

### Analysis of the obesity susceptibility loci in Qataris

The 23 obesity susceptibility loci selected for this study are listed in Additional file 1: Table S1.

Out of the 23 SNPs assessed, only two were significantly associated to obesity in Qataris, namely rs987237 and rs10938397. No deviations from Hardy-Weinberg equilibrium were detected.

The association of rs987237 and rs10938397 SNPs with obesity is shown in Table 2 and Table 3, respectively. Only lean and obese individuals were included in the analysis and tested under three models, namely: unadjusted, adjusted for age and adjusted for age and gender.

**Table 2:** **Multiple logistic regression models for obesity for the rs987237 SNP**

**Models**	**Rs987237 (n = 673)**			
	AA, n = 459*	**AG, n = 187**	**GG, n = 27**	**P-trend**
	**Obese n = 341**	**Obese n = 130**	**Obese n = 11**	
Model 1	1	0.79 (0.54-1.15)	0.24 (0.11-0.53)	0.0014
Model 2	1	0.65 (0.42-1.01)	0.20 (0.07-0.56)	0.0029
Model 3	1	0.65 (0.42-1.01)	0.21 (0.08-0.57)	0.0031

Model 1: unadjusted; Model 2: adjusted for age; Model 3: adjusted for gender + Model 2.

A allele versus G allele, chi-square = 10.3; P = 0.0013.

*The analysis of one sample among the cases failed for rs987237 SNP.

**Table 3:** **Multiple logistic regression models for obesity for the rs10938397 SNP**

**Models**	**Rs10938397 (n = 674)**			
	**AA, n = 302**	**AG, n = 292**	**GG, n = 80**	**P-trend**
	**Obese n = 206**	**Obese n = 210**	**Obese n = 67**	
Model 1	1	1.19 (0.84-1.70)	2.40 (1.27-4.56)	0.0266
Model 2	1	1.02 (0.68-1.54)	1.67 (0.82-03.40)	0.3532
Model 3	1	1.06 (0.70 -1.61)	1.65 (0.81-3.36)	0.3885

Model 1: unadjusted; Model 2: adjusted for age; Model 3: adjusted for gender + Model 2.

A allele versus G allele, chi-square = 6.15; P = 0.013.

The frequencies of rs987237 A and G alleles were respectively 0.77 and 0.23 in lean and 0.84 and 0.16 in obese subjects.

The frequencies of rs10938397 A and G alleles were respectively 0.72 and 0.28 in lean and 0.64 and 0.36 in obese subjects.

**Figure 1:**
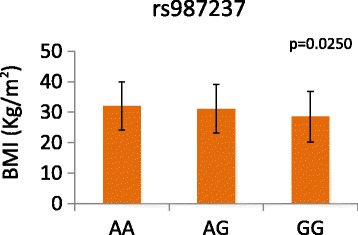
BMI distribution of the AA, AG and GG genotypes for the rs987237 SNP. Error bars indicate standard deviation.

When evaluating the association of rs987237 to obesity under the model adjusted for age and gender, the GG genotype was associated with a protective role (GG versus AA, P = 0.0031). This observation contrasts with findings from other reports which show a predisposing role in populations with different ethnicities [9,22]. The AA, AG and GG genotypes showed a gradient in BMI for the rs987237 (Figure 1).

**Figure 2:**
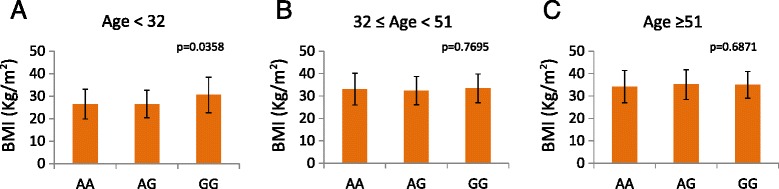
BMI distribution of the AA, AG and GG genotypes for the rs10938397 SNP according to the age tertiles: age < 32 y **(A)**, 32 y ≤ age < 51 y **(B)**, age Age ≥ 51 y **(C)**. Error bars indicate standard deviation.

In agreement with previous reports, rs10938397 GG genotype showed a predisposing effect with an almost 2-fold increased risk of obesity in GG carriers compared to the AA genotype. Although this association did not reach statistical significance [9,22,24], when breaking down all the individuals tested in age tertiles (< 32 years, ≥ 32 years and < 51 years, ≥ 51 years), the association became significant in the youngest group (age < 32 years, p = 0.0358) (Figure 2).

Additional file 1: Tables S2 and S3 show the distribution of rs987237 and rs10938397 genotypes, respectively according to the specific clinical and phenotypic variables.

### The genetic signature of the Qatari population as defined by the 23 obesity SNPs

We compared the allele distribution of the 23 obesity SNPs in lean Qatari population with that of the 1000 Genomes Project.

**Figure 3:**
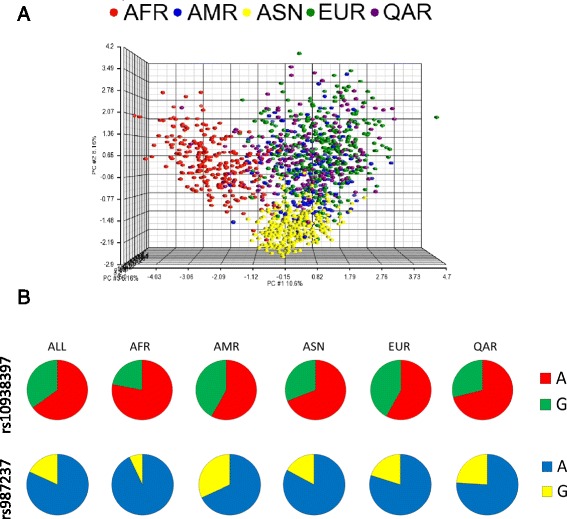
Comparison of African (AFR), Asian (ASN), American (AMR), European (EUR) and Qatari (QAR) population data. Principal component analysis (PCA) based on the 23 obesity loci **(A)**. Distribution of rs987237 and rs10938397 allelic frequencies among World populations **(B)**.

Albeit these 23 SNPs were able to discriminate the major genetic groups (African, Asian and Caucasian), we found a general admixture of Qatari individuals with subjects of European as well as African and Asian origin although to a lesser extent (Figure 3A). This could reflect the different tribal ancestries of the Qatari population.

As for the only two SNPs which turned out to be significant in the Qatari population, their frequency was different from the frequencies of the other populations (Figure 3B).

## Discussion

Obesity has become a major global health problem together with all the complications and diseases associated with body fat accumulation. Although the increasing incidence and prevalence of obesity can be predominantly explained by lifestyle changes (high intake of fast food and less physical activity), yet some individuals seem to be more susceptible to obesity than others highlighting an important genetic component. Unfortunately, to date no studies are available to dissect the genetic component of obesity in the Qatari population. In the current study we evaluated whether previously established obesity loci associate to obesity also in the Qatari population. Toward this aim, we selected 23 SNPs which have been related to BMI and waist circumference in previous GWAS studies [9,10,18-22]. These SNPs were chosen as representative of the obesity status in Western populations (as no comprehensive studies are available yet for the Arabian populations) and because close or within genes whose association to obesity has been well established (e.g., FTO, MC4R). Studying the effect of these 23 SNPs becomes important considering that little is known about the predisposition to obesity in Qataris.

Unexpectedly, only 2 of the 23 SNPs tested were significantly associated with obesity, namely rs987237 and rs10938397.

The rs987237 SNP maps in the TFAP2B gene. TFAP2B encodes a transcription factor, which is expressed preferentially in adipose tissue. Its role in regulating the adipocyte function and adipokine expression is believed to be responsible for the functional link to obesity [22,25]. It has been shown that overexpression of TFAP2B results in lipid accumulation by enhancing glucose transport and inducing insulin resistance [25,26] and decreases the secretion of adiponectine and leptin in human adypocites [25,27]. The TFAP2B rs987237 SNP has been found associated with WC and BMI in a meta-analysis study which included 16 GWAS of subjects of European ancestry [22]. In the present study, the rs987237 G allele showed a significant association with protection to obesity, however the direction of the effect was opposite to that reported initially [9,22]. Opposite direction on the effect of a given allele has been observed in several studies either among different populations or within the same population [28,29]. In the case of rs987237, the opposite direction has been reported in a recent study from Albuquerque and colleagues which showed a nominal association of the rs987237 A allele with the risk of obesity in a population of Portuguese children [30]. Further studies with a larger sample size of Qatari individuals are needed to confirm these findings.

As for rs10938397 SNP, we found a significant contribution of the minor allele of rs10938397 to increased risk of obesity in individuals of age < 32 years. This SNP maps near GNPDA2 gene, which encodes the glucosamine-6-phosphate isomerase, an enzyme involved in the carbohydrate metabolism. The association between GNPDA2 and BMI has firstly been identified in a meta-analysis of several European GWAS performed by the GIANT consortium [10,24]. Subsequently, the association has been confirmed in other populations of adults from East-Asia and of Chinese population [31,32]. However, our study is the first to evaluate the association of this SNP in Qataris. Notably, the association reached significance only in individuals of age < 32 years. The reason behind the age-related association is not clear, lifetime environmental and/or body composition changes may explain such phenomenon. The findings from a recent study of the association of rs10938397 in Chinese children support our findings and provide rationale for further investigation [33]. Alternatively, it is possible that the sample size of our cohort did not have enough power to detect association in older individuals. Further studies are necessary to validate our results.

With regards to the other variations, it should be stated that the fact that we did not find association with SNPs within or near well-known obesity predisposing genes (i.e., FTO and MC4R) might indicate that loci other than the ones we tested in our study might have a role in predisposing to obesity in Qataris.

Targeted replication studies of FTO gene in African and African Americans have yielded inconsistent results, potentially due to the difference in linkage disequilibrium (LD) structure between FTO SNPs in African compared with European or Asian ancestry populations, as reviewed by Loos RJF and Yeo GSH [34].

Although FTO is most probably the strongest and best replicated obesity gene [7,34], different GWAS studies for obesity-related traits in populations with European ancestry identified different FTO SNPs [20,21,34], with rs9939609 most commonly associated to obesity. All GWAS-identified FTO SNPs belong to the same highly correlated cluster, which covers about 46 kb in the first intron of FTO (including rs1558902). The cluster of BMI-associated FTO SNPs in East Asian populations is very similar to that of European ancestry populations. However, in populations of African ancestry, the correlation between SNPs in the first intron of FTO is much weaker than in those with European ancestry (Additional file 2: Figure S1). As we do not know much about the LD architecture of the Qatari genome, we cannot exclude that rs1558902 SNP may be in a different LD block from the one that includes the real FTO functional variation.

Interestingly, concordant to our findings, a study from Shimaoka and colleagues failed to demonstrate statistical association of the FTO rs1558902 SNP in a cohort of almost one thousand five hundred Japanese subjects [35]. Furthermore, rs1421025 SNP located in intron 1 of the FTO gene (as rs1558902 tested in this study) showed absence of association to obesity in Mexican children [24].

Replication of the association of these 23 SNPs in Qataris is not obvious and ethnic differences in linkage disequilibrium patterns, ethnicity-specific associations and gene-environment interactions may cloud the picture. Further additional studies should be performed to replicate our findings.

It is noteworthy that these 23 established genetic variations associated with obesity explain very little of the genetic risk for the Qatari population, suggesting the existence of additional loci whose number and effect size remain unknown and which should be the focus of future more intense investigation.

High-throughput technologies, including whole genome sequencing and chip-based genotyping on an extended number of lean and obese Qatari subjects would help in identifying additional loci and should be considered as a useful strategy.

## Conclusions

Our data support a different genetic profile associated with obesity in the Qatari population compared to Western populations, as only 2 among 23 SNPs have been found associated to obesity in the Qatari population. Studying the genetic background of Qataris is of primary importance as the etiology of a given disease might be population-specific.

## Additional files

Additional file 1: Table S1. List of the 23 obesity susceptibility loci. **Table S2.** Association of rs987237 SNP with phenotypic and clinical variables. **Table S3.** Association of rs10938397 SNP with phenotypic and clinical variables. Additional file 2: Figure S1. LD (r^2^) of the FTO SNPs within intron 1 retrieved from Ensembl (www.ensembl.org, location coordinates based on Ensembl 75 (GRCh37.p13), 16: 53783574-53823573).
