# Supplementary material for: Obesity susceptibility loci in Qataris, a highly consanguineous Arabian population
Source: J Transl Med. 2015 Apr 13;13:119. doi: 10.1186/s12967-015-0459-3 (PMC4422146; doi:10.1186/s12967-015-0459-3)
Supplement: Additional file 1: Table S1. — List of the 23 obesity susceptibility loci. Table S2. Association of rs987237 SNP with phenotypic and clinical variables. Table S3. Association of rs10938397 SNP with phenotypic and clinical variables. [file 12967_2015_459_MOESM1_ESM.docx]

**Supplementary Table 1**. List of the 23 obesity susceptibility loci

| **SNP ID** | **Nearby gene** | **Chr.** | **Position^a^ (bp)** | **Alleles** |
| --- | --- | --- | --- | --- |
| rs1558902 | FTO | 16 | 53803574 | T/A |
| rs2867125 | TMEM18 | 2 | 622827 | T/C |
| rs571312 | MC4R | 18 | 57839769 | C/A |
| rs10938397 | GNPDA2 | 4 | 45182527 | A/G |
| rs2815752 | NEGR1 | 1 | 72812440 | G/A |
| rs7359397 | SH2B1 | 16 | 28885659 | C/T |
| rs9816226 | ETV5 | 3 | 185834499 | A/T |
| rs3817334 | MTCH2 | 11 | 47650993 | C/T |
| rs987237 | TFAP2B | 6 | 50803050 | A/G |
| rs7138803 | FAIM2 | 12 | 50247468 | G/A |
| rs10150332 | NRXN3 | 14 | 79936964 | T/C |
| rs2287019 | QPCTL | 19 | 46202172 | C/T |
| rs13107325 | SLC39A8 | 4 | 103188709 | C/T |
| rs3810291 | ZC3H4 | 19 | 47569003 | G/A |
| rs2890652 | LRP1B | 2 | 142959931 | T/C |
| rs887912 | FANCL | 2 | 59302877 | T/C |
| rs13078807 | CADM2 | 3 | 85884150 | A/G |
| rs11847697 | PRKD1 | 14 | 30515112 | C/T |
| rs1555543 | PTBP2 | 1 | 96944797 | A/C |
| rs4771122 | MTIF3 | 13 | 28020180 | G/A |
| rs4836133 | ZNF608 | 5 | 124332103 | A/C |
| rs4929949 | RPL27A | 11 | 8604593 | T/C |
| rs543874 | SEC16B | 1 | 177889480 | A/G |

Nearby gene is the closest to the SNP in column 1. Chr., chromosome.

^a^Positions according to Genome Reference Consortium human genome (build 37) and allele coding based on the positive strand.

**Supplementary Table 2**. Association of rs987237 SNP with phenotypic and clinical variables

| **Variable** | **AA** | | **AG** | | **GG** | **p-value** |
| --- | --- | --- | --- | --- | --- | --- |
| N* | 317 | 119 | | 18 | |  |
| BMI (kg/m^2^) | 29.9±7.1 | 29.6±6.4 | | 27.0±8.0 | | 0.22 |
| WC (cm) | 95.0±16.3 | 93.8±15.0 | | 90.53±17.1 | | 0.43 |
| CVD (Y/N) | 67/250 | 22/97 | | 2/16 | | 0.52 |
| T2DM (Y/N) | 68/249 | 17/102 | | 3/15 | | 0.23 |
| Cholesterol disorders (Y/N) | 78/239 | 28/91 | | 6/12 | | 0.66 |
| Sleep hours (A/B/C) | 89/181/47 | 41/61/17 | | 5/12/1 | | 0.53 |
| Sleep disorder (Y/N) | 97/220 | 37/82 | | 4/14 | | 0.74 |

*Complete data were available for 454 subjects.

BMI, body mass index; WC, waist circumference; Sleep hours (A/B/C), A, less than 6 hours, B, between 6 and 8 hours, C, more than 8 hours; Y, yes (presence); N, no (absence); CVD, cardiovascular diseases; T2DM, type 2 diabetes mellitus; ANOVA and chi-squared test were applied as appropriate.

**Supplementary Table 3**. Association of rs10938397 SNP with phenotypic and clinical variables

| **Variable** | **AA** | | **AG** | | **GG** | **p-value** |
| --- | --- | --- | --- | --- | --- | --- |
| N* | 217 | 195 | | 42 | |  |
| BMI (kg/m^2^) | 29.5±7.4 | 29.7±6.6 | | 31.2±6.5 | | 0.34 |
| WC (cm) | 93.6±15.9 | 95.0±15.6 | | 97.2±18.1 | | 0.36 |
| CVD (Y/N) | 43/174 | 35/160 | | 13/29 | | 0.16 |
| T2DM (Y/N) | 42/175 | 37/158 | | 9/33 | | 0.93 |
| Cholesterol disorders (Y/N) | 49/168 | 49/146 | | 14/28 | | 0.33 |
| Sleep hours (A/B/C) | 63/124/30 | 61/106/28 | | 11/24/7 | | 0.95 |
| Sleep disorder (Y/N) | 67/150 | 62/133 | | 9/33 | | 0.41 |

*Complete data were available for 454 subjects.

BMI, body mass index; WC, waist circumference; Sleep hours (A/B/C), A, less than 6 hours, B, between 6 and 8 hours, C, more than 8 hours; Y, yes (presence); N, no (absence); CVD, cardiovascular diseases; T2DM, type 2 diabetes mellitus; ANOVA and chi-squared test were applied as appropriate.
