# Supplementary material for: Obesity susceptibility loci in Qataris, a highly consanguineous Arabian population
Source: J Transl Med. 2015 Apr 13;13:119. doi: 10.1186/s12967-015-0459-3 (PMC4422146; doi:10.1186/s12967-015-0459-3)
Supplement: Additional file 2: Figure S1. — LD (r2) of the FTO SNPs within intron 1 retrieved from Ensembl (www.ensembl.org, location coordinates based on Ensembl 75 (GRCh37.p13), 16: 53783574-53823573). [file 12967_2015_459_MOESM2_ESM.pptx]

## Slide 1
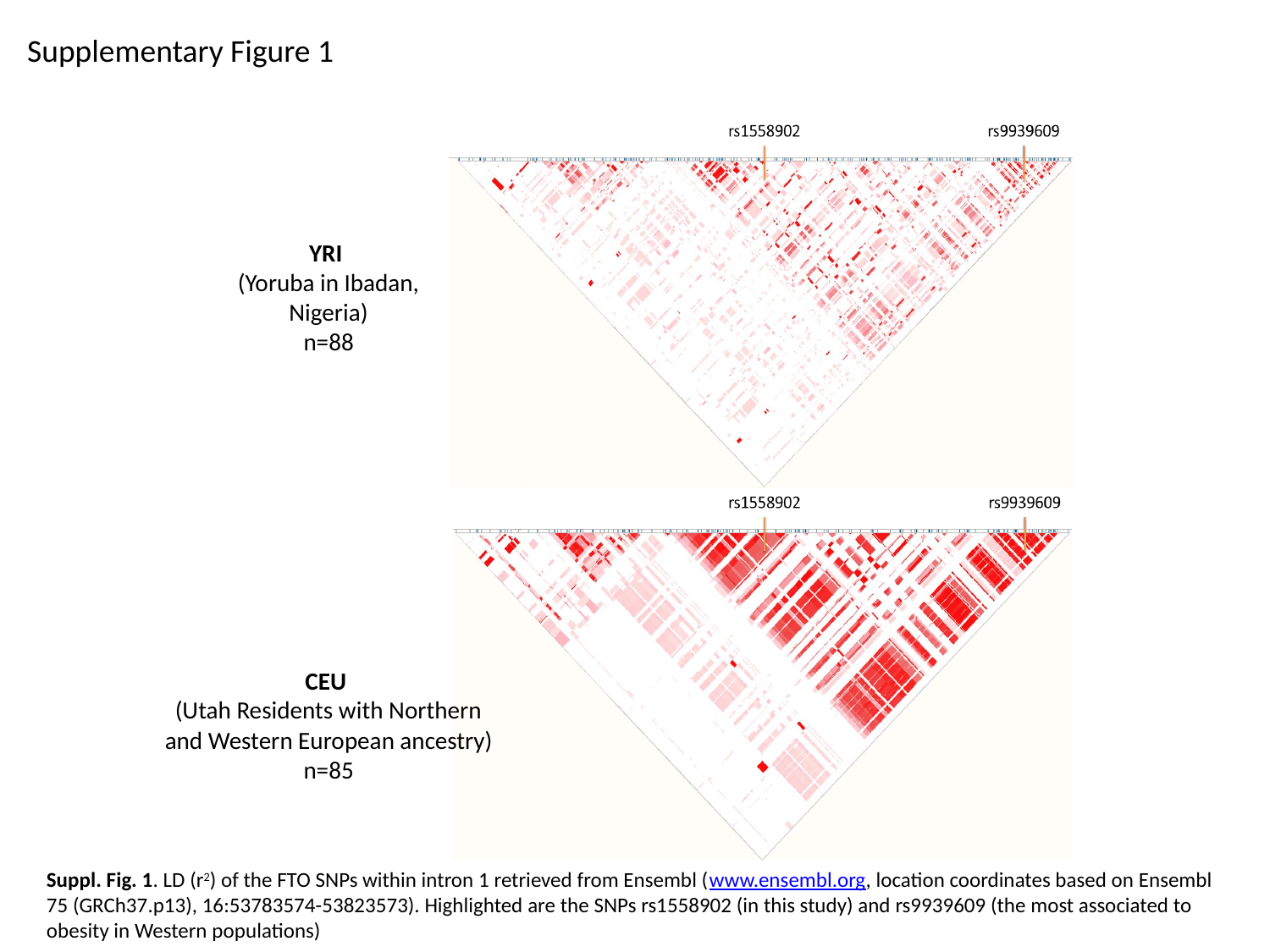

Supplementary Figure 1
YRI
(Yoruba in Ibadan, Nigeria)
n=88
CEU
(Utah Residents with Northern and Western European ancestry)
n=85
Suppl. Fig. 1. LD (r2) of the FTO SNPs within intron 1 retrieved from Ensembl (www.ensembl.org, location coordinates based on Ensembl 75 (GRCh37.p13), 16:53783574-53823573). Highlighted are the SNPs rs1558902 (in this study) and rs9939609 (the most associated to obesity in Western populations)
